# Resistance to Integrase Inhibitors

**DOI:** 10.3390/v2071347

**Published:** 2010-06-25

**Authors:** Mathieu Métifiot, Christophe Marchand, Kasthuraiah Maddali, Yves Pommier

**Affiliations:** Laboratory of Molecular Pharmacology, Center for Cancer Research, National Cancer Institute, National Institutes of Health, 37 Convent Drive, Bethesda, MD 20892, USA; E-Mails: metifiotma@mail.nih.gov (M.M.); marchanc@mail.nih.gov (C.M.); maddalik@mail.nih.gov (K.M.)

**Keywords:** AIDS, HIV-1 integrase, Raltegravir, Elvitegravir, GSK-1349572, GSK-1265744, interfacial inhibitors, resistance

## Abstract

Integrase (IN) is a clinically validated target for the treatment of human immunodeficiency virus infections and raltegravir exhibits remarkable clinical activity. The next most advanced IN inhibitor is elvitegravir. However, mutant viruses lead to treatment failure and mutations within the IN coding sequence appear to confer cross-resistance. The characterization of those mutations is critical for the development of second generation IN inhibitors to overcome resistance. This review focuses on IN resistance based on structural and biochemical data, and on the role of the IN flexible loop *i.e.,* between residues G140-G149 in drug action and resistance.

## 1. Background

**Figure 1:**
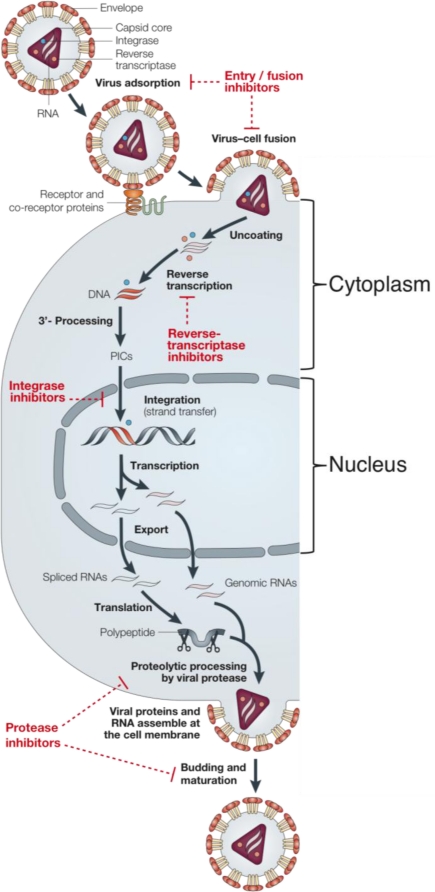
HIV-1 life cycle. HIV-1 first binds cells via interactions between the viral glycoproteins gp120/gp41 and the cellular receptor CD4 and CCR5 or CXCR4 co-receptors. After a conformational change of the complex, gp41 allows the fusion of both viral and cellular membranes, leading to the release within the cytoplasm of the viral core. The viral RNA (two copies of single-stranded RNA) is reverse-transcribed by the viral reverse transcriptase (RT) into double-stranded DNA. This DNA is then processed by the viral integrase (IN) and organized within a large nucleoprotein complex, the pre-integration complex (PIC). Following nuclear translocation, IN catalyzes the integration of this same DNA within the host genome (strand transfer, ST). After transcription, the viral RNA can be exported as genomic RNA (non-spliced) or as mRNAs (spliced RNA). The spliced RNA is translated into viral polyproteins that are cleaved by the viral protease (PR) and associate with genomic RNA at the cell membrane. Maturation by PR leads to new infectious particles released from the infected cell by budding. Drug targets include four different steps of viral replication: entry/fusion, reverse transcription, integration, and maturation (red). (Adapted from [3]).

During replication of the human immunodeficiency virus type 1 (HIV-1, Figure 1), integrase (IN) plays key roles at various steps of the replicative cycle [1]. In addition to its integration activity, IN interacts with the viral reverse transcriptase (RT) and can regulate the reverse transcription process [2].

**Figure 2:**
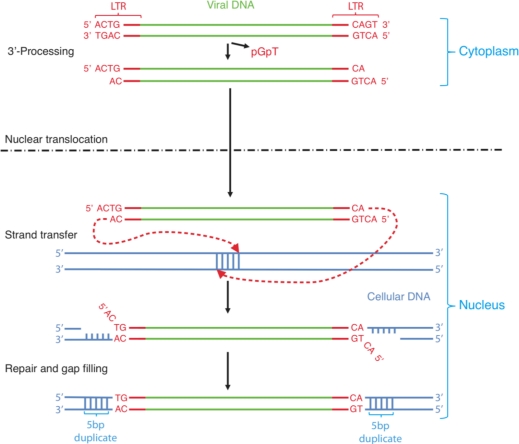
The integration process. IN catalyzes the integration of the HIV DNA within the cellular DNA in two distinct steps. First, IN cleaves the dinucleotide GT at both 3′ extremities of the viral DNA (red) by 3′-processing (3′-P). After nuclear import, the strand transfer (ST) reaction leads to the integration of the viral DNA (green) into the cellular DNA (blue). Cellular proteins then repair the newly created junctions, cleave the overhangs and fill the gaps, duplicating five bases of the cellular DNA on each side.

For this reason, mutations within the IN coding region of the *pol* gene can alter not only integration but also other steps in virus replication. Here, we will only focus on the reactions catalyzed directly by IN (Figure 2).

IN catalytic activity takes place following reverse transcription (Figure 1), as it associates with the long terminal repeats (LTR) of the newly synthesized viral DNA ends at the motif CAGT (Figure 2) [3–5]. A water molecule is used as the nucleophile to cleave the terminal dinucleotide GT. This first transesterification, 3′-processing (3′-P), takes place in the cytoplasm of the infected cell and is catalyzed by at least a dimer of IN [6] within a large nucleoprotein complex, the pre-integration complex (PIC), which includes viral and cellular co-factors in addition to IN and the reverse-transcribed viral DNA [7,8]. The PIC migrates to the nucleus via the microtubule network and through a nuclear pore [9]. In the nucleus, the PIC targets the host DNA mainly in transcribing regions. This targeting is directed by cellular co-factors such as LEDGF/p75 [10–12]. The integration of both viral DNA ends, or concerted integration, occurs with a five base pair stagger on opposite strands of the genomic DNA (Figure 2) [4]. This second transesterification, also called strand transfer (ST), uses the free 3′-OH extremity of the viral DNA as the nucleophile to attack the target DNA within at least a tetramer of IN [6]. The final process of integration is the repair of the junctions between the viral and host DNA, probably by cellular proteins. Both 3′-P and ST activities can be reproduced biochemically with recombinant IN and oligonucleotides mimicking the viral LTR [13–15].

## 2. Integrase structure

**Figure 3:**
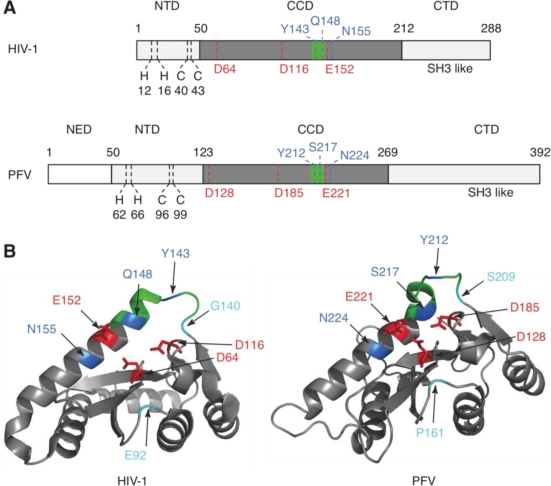
HIV-1 and PFV IN structures. **A.** Comparison of the primary structures of HIV-1 and PFV IN. N-terminal (NTD) and C-terminal domains (CTD) are represented in light gray and catalytic core domains (CCD) in dark gray. The DDE motif is colored in red and mutations conferring resistance to RAL (positions 143, 148 and 155 for HIV-1; 212, 217 and 224 for PFV IN) are highlighted in blue. The flexible loop, comprising amino acids 140–149 for HIV-1 IN or 209–218 for PFV IN, is colored in green. **B.** Three-dimensional structure of HIV-1 and PFV IN core domains. Colors correspond to scheme A. In addition, amino acids 92 and 140 for HIV-1 IN and 161 and 209 for PFV IN are highlighted in light blue. Cartoon representations were obtained using MacPyMol version 0.99rc6 and the pdb file 2B4F (HIV-1 IN core domain, residue 57–207, with mutations F185K) and 3L2R (PFV IN complete structure with viral DNA and Mg/Zn cations, residue represented 123–269).

IN is a 32 kDa protein. It belongs to the nuclease-transposase superfamilly including RNase H, Ruv C, transposases and other retroviral integrases. HIV-1 IN contains 288 amino acids forming three domains (Figure 3A). The N-terminal domain (NTD) contains amino acids 1–49 and a zinc-binding motif H_12_H_16_C_40_C_43_ involved in the oligomerization of IN. The central catalytic core domain (CCD) contains amino acids 50–212 and harbors the catalytic DDE motif (D_64_D_116_E_152_) well conserved among the retroviral integrase superfamily [16]. This triad coordinates two metal co-factors required for DNA binding. In biochemical assays, the recombinant enzyme can use either Mn^2+^ or Mg^2+^ but Mg^2+^ is the likely physiological cation. The C-terminus domain (CTD) contains amino acids 213–288 and carries a SH3-like domain implicated in DNA binding.

Even if the structure of the whole protein has not been resolved yet, several crystals of the isolated domains [17–19] or combinations of two domains of IN [20,21] have been obtained. All three domains of IN form homodimers in solution. They are also all involved in the binding of both viral and cellular DNA. Those crystal structures and electron microscopy have allowed the modeling of the global shape of HIV-1 IN [22,23].

The catalytic site of HIV-1 IN has still not been resolved in the presence of DNA. Moreover, a flexible loop close to the active site, comprising amino acids 140–149, with poor diffraction properties, was originally only resolved when mutated (G140A+G149A) [18]. A crystal containing a dimer of the IN catalytic core in complex with IBD (IN Binding Domain of LEDGF/p75) showed the structure of the WT flexible loop in one of the IN subunits (Figure 3B) [24]. Recently, an NMR structure of a dimer of the core IN provided further information on the flexible loop structure [25]. Using chemical crosslinking and modeling, the flexible loop, which is crucial for IN activity appears to be in close contact with the non-cleaved strand of the viral DNA (GTCA-5′, Figure 2) [23,26–30] and residues Y143 and Q148 interact with the 5′-terminal CA overhang [23,26,27,30]. Based on modeling of the murine leukemia virus (MLV) IN, the flexible loop has been proposed to open and stabilize the 5′ overhang of the double-stranded viral DNA extremity to allow ST after 3′-P [31].

**Figure 4:**
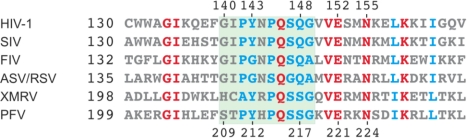
Amino acid alignment of the flexible loop region from different retroviruses. Alignment of the amino acid sequences corresponding to the region around the flexible loop of HIV-1, simian immunodeficiency virus (SIV), FIV, avian/Rous sarcoma virus ASV/RSV, xenotropic murine leukemia-related retrovirus (XMRV) and PFV. The flexible loop region is shaded in green. Residues identical in all the retroviruses are colored in red, and conserved residues with only one amino acid difference existing between the retroviruses are colored in blue. Numbers refer to the first residue for each sequence. Numbering at the top of the alignment corresponds to HIV-1 IN residues involved in resistance to RAL and to the catalytic glutamic acid (E152). Numbering for PFV IN is reported underneath the alignment.

Very recently, the complete structure of the prototype foamy virus (PFV) IN has been resolved in complex with a short oligonucleotide substrate that mimics the viral DNA end after 3′-P, providing the first three-dimensional structure of an active IN active site [32]. PFV IN differs from HIV-1 IN by the presence of an N-terminal extension domain (NED), which is conserved among the spumaviral and gammaretroviral INs (Figure 3A) [4,33]. This extra domain is implicated in non-specific DNA binding to the phosphodiester backbone of the viral LTR. The rest of the PFV IN protein is globally very similar to HIV-1 IN (Figure 3). The integration signature of PFV IN is different from that of HIV-1 IN (4 base pair duplication for PFV instead of 5 for HIV) [33]. Nevertheless, raltegravir and elvitegravir also inhibit PFV IN *in vitro* [33], indicating that the active site of the PFV and HIV enzymes are quite similar. Several crystal structure of PFV IN are now available with either Mg^2+^ or Mn^2+^ and without a metal co-factor (apo-enzyme) [32]. They show the importance of the flexible loop in binding and distorting the viral DNA to allow the attack of the 3′ LTR extremity on the target DNA during ST. Also, the flexible loop appears involved in RAL and EVG binding, highlighting a crucial role of the flexible loop in both IN activity and drug resistance [32]. From examination of the flexible loop region of several retroviral INs, PFV IN appears close to HIV-1 IN (Figure 4) with six out of 10 identical amino acids. A recent NMR study using the HIV-1 IN core domain showed that the HIV-1 and PFV INs flexible loops are highly similar [25]. The similarities between PFV and HIV legitimate the use of PFV IN as a model for HIV-1 IN.

## 3. Integrase inhibitors: historical overview

Using biochemical assays, several classes of IN inhibitors have been discovered over the past 17 years [1,34–36]. Hydroxylated natural products like dihydroxynaphtoquinones, isoflavones, chicoric acid and caffeic acid were reported in early biochemical studies [14,37]. Derivatives were developed (CAPE, 5-CITEP, V-165) and the most important family of inhibitors that emerged was the diketo acids (DKA) [38]. L-870,810 (Merck & Co.) and S-1360 (Shionogi & Co. Ltd. and GlaxoSmithKline) were the first DKA-like IN inhibitors to reach clinical trials. DKA derivatives are ST-selective inhibitors (INSTI) with high specificity for IN-DNA complexes and antiviral activity. Clinical trials with S-1360 and L-870,810 were terminated in phase I/II and II due to limited efficacy and toxicity, respectively [34,35]. Peptides and nucleic acid inhibitors of IN *in vitro* have also been identified by screening (phage display, yeast 2-hybrids, SELEX) or rational design (derived from viral or cellular co-factors) [39,40]. Zintevir (AR177) (Aronex Pharmaceuticals) is a G-quadraduplex-forming oligonucleotide inhibitor of both recombinant IN and HIV-1 replication at low nanomolar concentration [41,42]. It entered clinical trials in 1996, before the DKA, but was shown to target viral entry in cells and clinical trials were discontinued [43,44].

## 4. Raltegravir

After years of sustained effort, Merck and Co. successfully developed raltegravir (RAL, Isentress® also known as MK-0518), which was approved by the FDA in late 2007 as the first IN inhibitor (Table 1). As for DKA, RAL’s inhibition mechanism is specific for the ST step of integration and proposed to involve chelation of one or two metals within the IN active site after processing of the bound viral DNA ends [45,46].

**Figure 5:**
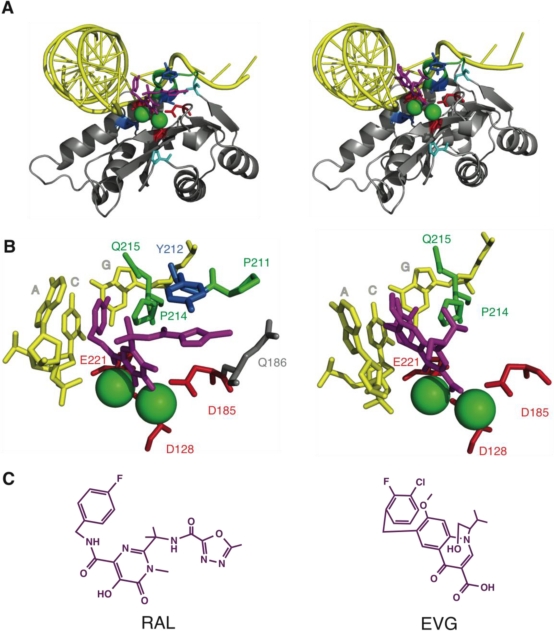
Crystal structures of PFV IN in complex with INSTI. **A.** Three-dimensional structure of the PFV IN core domain in complex with its viral DNA substrate and RAL (left) or EVG (right) in the presence of magnesium. The core domain (amino acids 123–269) is colored in grey. Positions 212, 217 and 224, corresponding positions 143, 148 and 155 in HIV-1 IN are highlighted in blue. Positions 161 and 209, corresponding to secondary mutation positions 92 and 140 for HIV-1 IN, are highlighted in cyan. Cartoon representations were obtained using MacPyMol version 0.99rc6 and the pdb files 3L2T (RAL) and 3L2U (EVG) containing the PFV IN complete structure with viral DNA and Mg^2+^/Zn^2+^. **B.** Close view of the active site containing RAL (left) or EVG (right). Only the residues of PFV IN and the bases of the viral DNA within 5 Å of the drug are represented as sticks. Colors are similar to panel A. **C.** RAL (left) and EVG (right) chemical structures oriented as in the PFV IN structure in panel B.

Crystal structures obtained recently with RAL bound to the full length PFV IN confirmed the two metal binding mode (Figure 5A) [32]. Binding of RAL also induces a displacement of the viral DNA within the IN active site, moving the terminal 3′-A of the conserved CA by more than 6 Å from its original position [32]. RAL also makes Van Der Waals (VDW) interactions with the conserved CA dinucleotide and the fourth guanine from the end of the non-cleaved strand (base paired with the conserved C) (see Figure 2). In addition, RAL contacts several amino acids, including P214 and Q215 (corresponding to P145 and Q146 for HIV-1 IN, respectively) by VDW interactions, and P214 (P145) by hydrophobic interaction and Y212 (Y143) by stacking interaction. RAL also forms polar interactions with the catalytic triad (DDE) and the two Mg^2+^ (Figure 5B). This mode of binding is consistent with the interfacial inhibition paradigm [3,51] whereby RAL was proposed to bind at the interface of IN and its DNA substrate following 3′-P.

First used in regimen of heavily treated patients, RAL is now approved (since July 2009) for first line therapy in combination with other drugs used in the highly active anti-retroviral therapy (HAART) combination regimen [52–56]. RAL has a remarkably low toxicity, exhibits high potency and favorable pharmacokinetics. In first line therapy, the oral formulation (400 mg twice a day) induces a large decrease of viral RNA load below detection levels within a few weeks [57]. The main degradation pathway of RAL involves glucuronidation [48]. RAL’s half-life presents two phases: an initial phase with a one hour half-life followed by a final phase with 7–12 hours half-life. An ongoing trial is also investigating the potential of a once-daily use of RAL (NCT00745823).

## 5. IN mutations conferring RAL resistance

Resistant viruses to RAL emerge by mutations within the IN coding sequence, which clearly demonstrates that IN is the target of RAL. The biochemically characterized mutations are summarized in Table 2, and an extensive list of IN mutations is available at http://ccrod.cancer.gov/confluence/display/LMPDNA/Home. Noticeably, the pattern of IN mutations for RAL overlaps with the previously reported mutations for the DKA derivatives (Table 2) [38,58].

Primary mutations involve three independent and non-overlapping genetic pathways: mutation N155H, mutations Q148H/R/K and less frequently mutations Y143/R/C/H (Figure 3) [59,60]. These mutations also affect IN activity and viral replication (Table 2) [29,59,61,62]. Some mutations may involve integration-independent mechanisms. For example, the mutation Y143G has been reported to interfere with the completion of reverse transcription [62,63].

Additional mutations have been reported including V72I, L74M, E92Q, T97A, E138A/K, G140S/A, V151I, E157Q, G163R/K, D232N [64–68]. Natural polymorphism studies showed that E157Q is present at a frequency of about 4–5%, while other mutations are highly infrequent [64]. Allele-specific real-time PCR has been used to detect very low levels of variants [69] and specific primers to amplify mutations Q148H, Q148R or N155 (detection limit 0.1%, 0.1% and 0.05%, respectively). Before RAL exposure, the Q148R variant was detectable at low level (0.4% of the HIV pool) in most patients (81% in treatment experienced and 86% in naïve patients), but other mutations, Q148H and N155H, were not detectable [69].

Secondary mutations appear specifically for each primary mutation: secondary mutations at position E92 for either N155 or Y143, secondary mutation at position T97 for Y143, and secondary mutation at position G140 for Q148 (Table 2) [70]. These secondary mutations are likely to increase RAL resistance (E92) and/or restore defective IN activity and viral fitness (G140) resulting from the primary mutations (Figure 3) [59,61]. The double-mutation at positions 140 and 148 is the most common combination [59,70]. In patients, G140A tends to be associated with Q148R, and G140S with Q148H/K. Systematic biochemical analyses of recombinant IN for all the possible combinations demonstrated that only the G140S-Q148H combination within the same IN polypeptide can rescue the biochemical activity of IN and confer high resistance to RAL [71]. In agreement with these biochemical data, viruses harboring the double mutation G140S-Q148H exhibit high resistance to RAL (>1400-fold) and their replication capacity is close to WT (90% to 99%) (Table 2) [59,70]. For the primary mutation N155H, the secondary mutation E92Q markedly increases resistance (from 15–30-fold for N155H to around 500-fold for the double-mutant). However, it does not appear to rescue viral fitness, which remains approximately 30 to 50% of WT [59,70]. The third mutation pathway, involving position Y143, has been less investigated. It seems to markedly decrease IN activities and viral fitness [62]. While 3′-P is affected for both Y143R and Y143C, the ST activity of Y143R is less affected than for the Y143C mutant. Even if such mutant viruses appear highly resistant to RAL, their low replication capacity may explain their delayed occurrence in patients [62].

The order of appearance of mutant viruses may be related to two main parameters: the number of genetic changes needed to produce the specific mutant and the fitness/resistance of the mutants. Mutations G140S/A, Y143C/H, Q148H/R/K and N155H require only one nucleotide change. On the other hand, mutation Y143R and the double mutants G140-Q148 require two genetic alterations. Mutations at position 140 impair the ST activity (but not the 3′-P) without conferring resistance to RAL, and as a consequence do not present a selective advantage [71]. The mutation N155H leads to an active enzyme (and replicative virus) with an intermediate resistance to RAL (Table 2) [59]. This may explain why the pathway N155 appears first followed by the double-mutants 140–148 and the Y143R mutant. For mutations Y143C/H, only one substitution is required, but because of the defective IN *in vitro* activity and replication defect of the corresponding viruses [62], other mutations are probably required to rescue viral fitness and enhance resistance to RAL. The two mutations Y143C/H could also be a temporary path leading to Y143R, which has a better ST activity [62]. Even if mutations at position 143 can confer higher resistance to RAL than the double-mutant G140S-Q148H [62], the double-mutant G140S-Q148H presents a better enzymatic activity (almost at WT level *in vitro* and *in vivo*) [59,71,72], which may explain the prevalence of this double-mutation in RAL resistant viruses.

Recently, a prospective study of patients receiving RAL showed a link between RAL plasma concentration and the resistance pathway developed [73]. In patients developing resistance to RAL with mutation at position 155 and 143, the drug plasma concentration 12 h after tablet ingestion, corresponding to the lowest level of drug, was 50–100 ng/ml (100–200 nM) [73]. On the other hand, patients with mutations emerging at position 148 presented a higher level of RAL (300–350 ng/ml corresponding to 750–800 nM) [73]. RAL has a limited intestinal absorption [34] and resistance cannot be overcome by increasing dosing. Next generation drugs are warranted to overcome this pharmacological limitation and achieve high enough plasma concentrations to target RAL-resistant viruses.

## 6. Elvitegravir

Elvitegravir (EVG, GS-9137, JTK-303) is the next most advanced IN inhibitor. It is developed by Gilead Sciences and presently in phase III clinical trials (Table 1). *In vitro*, EVG exhibits potent anti-IN and anti-HIV activity at low digit nanomolar IC_50_ and EC_90_ [34,74]. EVG is a slightly more potent IN inhibitor than RAL [29,71]. However, it has also been reported to produce some non-specific toxicity in non-infected cells [34]. EVG is metabolized *in vivo* by cytochrome P450 and glucuronidation [48], and co-administration with ritonavir (PR inhibitor) increases EVG systemic concentrations by about 20-fold, consistent with a possible once daily use of the drug [75]. Ongoing phase III studies are investigating the potential of EVG dosed at 125 mg once daily, boosted with 85 mg of ritonavir (NCT00707733).

EVG resistance is associated with specific primary mutations such as T66I and S147G in addition to mutations common with RAL resistance (E92Q, Q148H/R/K and N155H) (Table 2) [64,67,76]. As for RAL, secondary mutations are selected under EVG exposure. Mutations H51Y, T66A/K, L68I/V, S119R/G, G140C, S153Y, K160N, R166S, E170A, S230R and R263K are specifically selected in addition to E138K, G140S, E157Q and D232N, which are common with RAL [67]. The resistance factor induced by the double-mutation G140S-Q148H is higher with EVG than with RAL [29,71]. Because EVG is slightly more potent than RAL (Table 1) and because its blood concentration can be boosted, it might be possible higher safe doses of EVG could still reach an active concentration window in patients developing mutant viruses.

Crystal structures obtained with the PFV IN show that EVG shares a common binding mode with RAL [32]. Similar to RAL, EVG binds as an interfacial inhibitor in the IN catalytic site. It presents VDW interactions with the conserved CA and the fourth G from the 3′-end of the non-cleaved strand and induces a displacement of the viral DNA. Regarding its drug-protein interactions, EVG exhibits closer interactions with P214 (corresponding to P145 in HIV-1 IN) [32]. Comparison of the RAL and EVG crystal structures shows that Y143, which harbors a stacking interaction with the oxadiazole ring of RAL, does not have as strong interaction with EVG (Figure 5B). Some *in vitro* data seems to confirm that IN issued from RAL resistant viruses harboring mutation Y143 as primary signature present a limited resistance to EVG (*i.e.,* smaller fold change) [73]. Thus, further studies are warranted to determine whether EVG remains active against the Y143 IN mutants that are selected by RAL exposure.

## 7. Second generation INSTI

New promising compounds are currently under phase II clinical trials [50]. Shionogi-GlaxoSmithKline (S-GSK) Pharmaceuticals, LLC (USA) is developing new IN inhibitors that should be active against RAL- and EVG-resistant IN mutants. The lead compound, GSK-1349572 displays low nanomolar IC_50_ *in vitro* and very long half-life in humans (14 h, Table 1). Similarly to RAL and EVG, GSK-1349572 inhibition involves the chelation of divalent ions within the active site through a two-metal binding scaffold [35,50]. The inhibition of viral replication (sub-nanomolar EC_50_) is specific for the integration step, with a total viral DNA remaining constant in the cell while the integrated DNA decreases and circular 2-LTR viral DNA increases [50]. GSK-1349572 seems to present good pharmacokinetics (Table 1). It is metabolized through UDP-glucoronosyltransferase (UGT) and its concentration can be decreased by certain multivitamin supplement or antacids [100]. Additionally, GSK-1349572 does not inhibit cytochrome P450 [50]. It also presents low inter-patient pharmacokinetics variability and reaches effective therapeutic concentrations even when used alone without boosting agent.

Regarding resistance mutations, GSK-1349572 is still in early development and highly resistant viruses have not been characterized. *In vitro* selection showed a different mutation pattern compared to RAL and EVG [50]. Resistance mutation selected under GSK-1349572 exposure comprised L101I, T124A, S153F/Y with four-fold resistance. When assessed against previously reported mutants resistant to RAL and EVG, GSK-1349572 did not appear to show cross-resistance [50]. For example, single mutants containing either E92Q, Q148H/R/K, S153Y or N155H and double-mutants containing E92Q-N155H or T97A-Y143R/C did not change the EC_50_ of the drug. However, the double-mutants G140S-Q148H/R exhibit 20-fold resistance to GSK-1349572. This improvement in term of resistance together with the slightly greater potency of GSK-1349572 compared to RAL may be sufficient for the drug to inhibit mutant virus replication.

S-GSK Pharmaceuticals is also developing in parallel a backup compound, GSK-1265744, which is currently in phase I/IIa. GSK-1265744 presents similar inhibitory and pharmacokinetic properties to GSK-1349572 with an increased half-life (30 h) [55].

## 8. Conclusions

Because of its central role in retroviral replication and of the effectiveness of RAL and EVG, IN is now a fully validated target for anti-HIV therapy. Before the approval of RAL, HIV-1 IN was not targeted with HAART. The good pharmacokinetics, minimal side effects and safety of RAL should allow its broad use in AIDS patients. EVG, the next most advanced IN inhibitor appears to share not only the same inhibition mechanism but also cross-resistance with RAL. It is likely that novel inhibitors such as the new drugs from S-GSK will overcome the RAL- and EVG-resistance mutations both by allowing more effective plasma concentrations and by acting at different sites of IN. The molecular characterization of resistant viruses is an essential step for the discovery and the development of new IN inhibitors.

## Figures and Tables

**Table 1:** INSTI currently in clinical trials.

**Name**	**Company**	**Structure**	**Anti-IN^a^**	**Anti-HIV^b^**	**T_1/2_**	**Status**	**Ref.**
Raltegravir MK-0518 Isenstress®	Merck & Co.	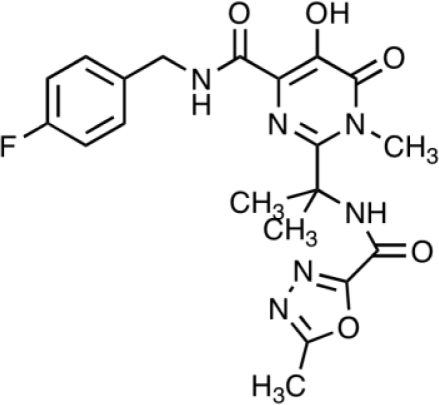	2–7	8.9	1; 7–12	FDA Approved October 2007	[35, 47–49]
Elvitegravir JTK-303 GS-9137	Japan Tobacco Inc. and Gilead Sciences	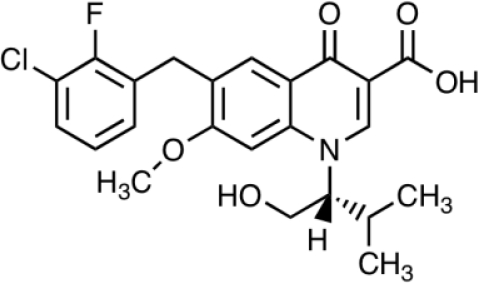	7	1.7	3; 9*	Phase III	[35, 47]
S/GSK-1349572	ViiV-Healthcare and Shionogi & Co. Ltd	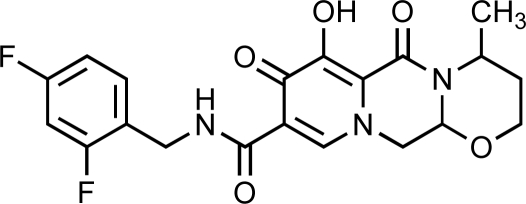	2.7	2	14	Phase IIb	[35, 50]

a IC_50_ and

b EC_90_ are expressed in nM. Half-life of compounds (T_1/2_) are expressed in h.

* When boosted with ritonavir.

**Table 2:** Characterized mutations within IN conferring resistance to RAL or EVG.

**Mutations**	**IN**			**Virus**		**Refs**
	**3′-P**	**ST**	**R^ce^**	**Selection**	**RC**	
H51Y		++		EVG		[76]
H51Y/E92Q/S147G		+		EVG		[76]
H51Y/E92Q/S147G/E157Q		++		EVG		[76]

T66A	+	++		RAL/EVG	++	[63,77,78]
T66I	+++*	+++*	5^1^–10^2^X	DKA/EVG	++*	[29,38,79–87]
T66I/E92Q		−		EVG		[85,86]

L74M	+++	+++	1X^1^,^2^	DKA/EVG		[29,80,84,85,88]

E92Q	+++*	+++*	4–5X^1^,^2^	DKA/RAL/EVG	++	[66,76,78,88]
E92Q/S147G		+		EVG		[76]
E92Q/N155H				RAL	++*	[72,89]

E138K/Q148HRK				DKA/RAL	++	[72,87,90]

G140S	+++*	+	1X^1^,^2^	DKA/RAL	++	[61,71,78,89–93]
G140A	++	+	2–3X^1^	RAL		[71]
G140S/Q148H	++*	+++*	>50X^1^,^2^	RAL	+++	[61,66,72,94]
G140S/Q148K	++	+	10–25X^1^	DKA/RAL	++	[71,72,90]
G140S/Q148R	−	+	>25X^1^	DKA/RAL	++	[56,72,87]
G140A/Q148H	−	+	>25X^1^	RAL	+	[71,72]
G140A/Q148K	−	−	2–3X^1^	RAL	++	[71,72]
G140A/Q148R	+	+	>25X^1^	RAL	++	[71,72,95]
G140S/Q148H/S230N				RAL	+++	[89]

Y143R	+	++	>50X^1^	RAL	+	[56,62,93,96]
Y143C	+	+	>50X^1^	RAL	+	[62]
Y143R/G163R				RAL	++	[89]

S147G		+		EVG		[76]

Q148K	−	+*	50X^1^*	DKA/RAL/EVG	+	[28,29,71,72,87,90]
Q148R	+*	+	2–3X^1^	DKA/RAL/EVG	++	[71,72,78,85–87,90,95,97]
Q148H	−	+*	2–3X^1^,^2^	RAL	++*	[61,71,72,78,89,93]
Q148H/N155H				RAL	+	[89]
Q148K/G163R				RAL		[87]

N155H	+++*	+++*	5–10X^1^,^2^	DKA/RAL	++	[29,61,66,72,78,86,87,89,93–95,97,98]

E157Q		++		RAL/EVG		[76,99]

S230R	+++	+++		DKA/EVG		[80,85]

**R^ce^**: resistance expressed as fold change in IC_50_; **RC**: replication capacity.

Signs refer to activity compared to WT: − = 0–10%;

+ = 10–40%;

++ = 40–80%;

+++ = 80–100%;

no entry = not tested.

1 RAL,

2 EVG,

* discordant data in literature (the highest value is presented).
